# The Hair

**Published:** 1891-10

**Authors:** 


### THE HAIR.

Hair is shed from various causes ; most of them are somewhat obscure, being of neurotic origin, and are not always amenable to treatment ; while in some cases the shedding is scarcely to be accounted a morbid process, but seems to be a kind of natural moult analogous to what is seen in birds and beasts which require a periodic change of fur or feathers. This human moult covers the shoulders and fills the comb with hair, but it soon rights itself and causes no real distress or trouble. The morbid causes of falling hair are faulty innervation, defective circulation, disordered skin, confined perspiration which keeps the head hot and sodden ; and among the minor causes may be enumerated too frequent washing with soap and hot water, too vigorous brushing with stiff scarifying brushes, and forcing the hair, by plaiting, pinning or otherwise, to maintain a position which is contrary to its natural drift. The natural drift or set of the human hair is easily indicated by laying a dessertspoon on the middle line of the head, with the handle pointing forwards and the bowl behind. The hairs trend away from both sides of the straight handle and from all around the circular bowl. Hence it is physiologically more correct to part the hair in the middle than at the side, which may contribute (among other causes) to the infrequency of baldness among women as compared with men. There is reason for believing that the structure of a hair is much more elaborate than is usually set down in text-books, and that the middle and inner rings have very definite functions to discharge, the inner ring, or rather core, having to supply the nutritive element, while the middle ring supplies the lubricant and pigment. Either of these may be disordered, producing in the one case loss of the hair itself, and in the other case loss of the color. A simple lotion which will do good in most cases and harm in none, is composed of 1 oz. of glycerine, 1 oz. of spirit of rosemary, in 10 oz. of water. Mix, and shake it well before using with a soft brush night and morning. With a little more trouble the efficacy of the lotion can be much increased by substituting for the 10 oz. of water a similar quantity of an infusion of the leaves of lemon-scented verbena—a favorite and common garden plant, otherwise known as Aloysia citriodora—which is made by pouring hot water on a jugful of the fresh green leaves, and letting it stand till cold. Then strain off the fluid, and pour it cold on a second lot of leaves. In an hour or two the fluid may be again strained off and used as above directed.

